# What Can Reinforcement Learning Models of Dopamine and Serotonin Tell Us about the Action of Antidepressants?

**DOI:** 10.5334/cpsy.83

**Published:** 2022-07-20

**Authors:** Denis C. L. Lan, Michael Browning

**Affiliations:** 1 Department of Experimental Psychology, University of Oxford, Oxford, GB; 2 Department of Psychiatry, University of Oxford, Oxford, GB

**Keywords:** serotonin, dopamine, depression, antidepressants, reinforcement learning

## Abstract

Although evidence suggests that antidepressants are effective at treating depression, the mechanisms behind antidepressant action remain unclear, especially at the cognitive/computational level. In recent years, reinforcement learning (RL) models have increasingly been used to characterise the roles of neurotransmitters and to probe the computations that might be altered in psychiatric disorders like depression. Hence, RL models might present an opportunity for us to better understand the computational mechanisms underlying antidepressant effects. Moreover, RL models may also help us shed light on how these computations may be implemented in the brain (e.g., in midbrain, striatal, and prefrontal regions) and how these neural mechanisms may be altered in depression and remediated by antidepressant treatments. In this paper, we evaluate the ability of RL models to help us understand the processes underlying antidepressant action. To do this, we review the preclinical literature on the roles of dopamine and serotonin in RL, draw links between these findings and clinical work investigating computations altered in depression, and appraise the evidence linking modification of RL processes to antidepressant function. Overall, while there is no shortage of promising ideas about the computational mechanisms underlying antidepressant effects, there is insufficient evidence directly implicating these mechanisms in the response of depressed patients to antidepressant treatment. Consequently, future studies should investigate these mechanisms in samples of depressed patients and assess whether modifications in RL processes mediate the clinical effect of antidepressant treatments.

## Introduction

Major Depressive Disorder (MDD) is a condition characterised by persistent low mood and reduced enjoyment, accompanied by symptoms like reduced concentration, energy, self-esteem, and altered appetite and sleep quality. More recently, researchers have focused on the association between MDD and reward processing deficits due to their potential to explain motivational deficits such as reduced interest and activity—for example, a meta-analysis of 48 case-control studies conducted by Halahakoon et al. (2020) found a consistent relationship between depression and reward-processing impairments such as reward bias and option valuation.

Evidence suggests that antidepressant drugs are moderately effective at treating MDD (Cipriani et al. 2018). Antidepressants typically work by modulating monoamine neurotransmitter function: for instance, selective serotonin reuptake inhibitors (SSRIs) increase synaptic serotonin levels by blocking the activity of serotonin transporters, while norepinephrine and dopamine reuptake inhibitors (NDRIs) increase synaptic norepinephrine and dopamine levels by blocking the action of norepinephrine and dopamine transporters.

Given that many antidepressants act to increase synaptic monoamine levels, early theories of antidepressant function supposed that depression was caused by deficiencies in synaptic concentrations of monoamine transmitters like serotonin and dopamine (Delgado, 2000). However, evidence for a direct role for synaptic concentrations of serotonin and dopamine in depression remains scarce (Cowen & Browning, 2015; Schneier et al., 2018). Instead, different lines of research have implicated several secondary processes, from molecular to cognitive, that better account for antidepressant effects. For example, some have proposed that the delayed onset of clinical effects following SSRI administration could be due to the time required for autoreceptors to be desensitised, a process that results in greater serotonin availability in the synapse (Artigas et al., 1996). Similarly, Andrews et al. (2015) posits that the serotonergic system serves the function of energy regulation, with elevated serotonin in depressed patients and following acute SSRI treatment supporting ruminative processes, and chronic SSRI treatment leading to compensatory responses to restore energy homeostasis that leads to the relief of symptoms. At the same time, other researchers have observed that antidepressant administration seems to increase neuroplasticity in animal models (e.g., Maya Vetencourt et al., 2008). Consequently, supporters of neuroplasticity models propose that depression is caused by compromised information processing within neuronal networks, and antidepressants work by restoring synaptic plasticity and allowing the brain to reshape its neuronal networks and restore normal information processing (Castrén, 2005). Focusing instead on the cognitive level of explanation, cognitive neuropsychological models propose that antidepressants work by increasing positive affective processing in depressed patients (Harmer, Goodwin, et al., 2009). These models suggest that although the increase in positive affective processing appears immediately after antidepressant administration (Harmer, O’Sullivan, et al., 2009), changes in mood may only become apparent after the patient relearns their emotional associations through the accrual of evidence influenced by the positive bias.

## Reinforcement Learning as a Possible Bridge between Levels of Explanation

Overall, the receptor desensitisation, neuroplasticity, and cognitive neuropsychological models of antidepressants are all relatively well-supported by empirical evidence, suggesting roles for both molecular (e.g., increasing synaptic monoamine availability or neuroplasticity) and environmental factors (e.g., in the relearning of emotional associations to reshape neuronal networks) in the action of antidepressants. However, the way in which antidepressants act to alter the interaction between these molecular and environmental factors remains unclear.

**Figure 1:**
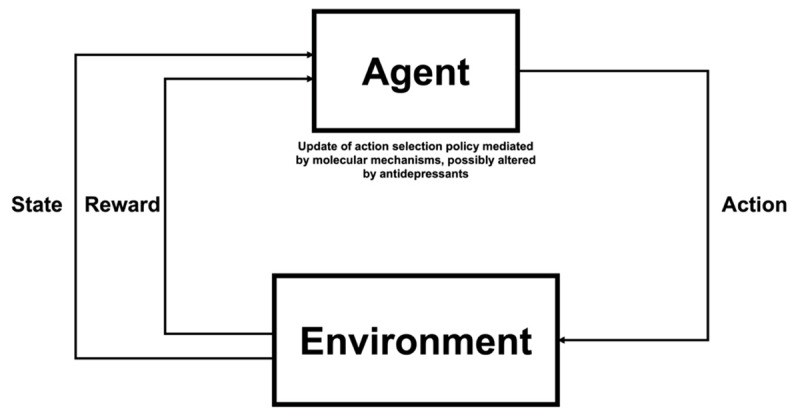
Schematic diagram illustrating the RL framework. At every time step, the agent receives information about the state they are in (i.e., a representation of their current environment, such as the speed and position of the car when driving) and the amount of reward they have received. Based on this feedback, the agent aims to adjust its action selection policy to maximise the amount of reward obtained in the future.

Computational models offer one way by which we might bridge these explanatory levels and thus link together the apparently distinct mechanistic accounts. One particularly successful class of model used in computational neuroscience is reinforcement learning (RL), which assumes that agents learn based on feedback from the environment to take actions that maximise the reward they obtain, as shown in Figure 1 below:

The presence of reward-processing deficits in depression makes RL a particularly attractive class of models for helping us probe the computations that are implicated in depression. In particular. RL models offer one way for us to characterise how the types of behaviours that depressed patients exhibit (i.e., ‘actions’ in RL models) may be determined by interactions between molecular mechanisms (that may play a role in the update of value estimates and action policies) and the environment and how these might be altered by antidepressant medication. In this paper, we review numerous ideas about how RL models may shed a light on antidepressant effects. We focus primarily on dopamine and serotonin—two neurotransmitters that are both commonly studied using the RL framework and are consistently implicated in the effects of antidepressants such as NDRIs and SSRIs. For each neurotransmitter, we first provide a high-level review of the potential roles that the transmitter plays in RL computations before evaluating the evidence linking these ideas with depression and, ultimately, antidepressant action.

## What is the Role of Dopamine in Reinforcement Learning?

### Dopamine as a ‘Reward Prediction Error’ Signal

#### Computational Background

In RL, the environment is typically conceived of as a Markov Decision Process (MDP), which can be defined by a set of states (*S*), transitions between states (*P*), a space of possible actions (*A*), and the rewards that can be obtained in each state (*R*). Given this formulation, the value for any given state under an optimal policy is given by the Bellman optimality equation:


1 \[
{V^*}\left( s \right) = {\mathrm{max}}\left\{ {{R_{t + 1}} + \gamma \cdot {V^*}\left( {{s_{t + 1}}} \right)} \right\}
\]

In other words, the optimal value of each state is given by maximising the sum of the reward obtained at the next timestep and the future rewards expected from traversing to the next state (discounted by a discount factor 0 ≤ *γ* ≤ 1). Note that in RL, it is conventional to use “*” to indicate the value of a variable under an optimal policy.

Although the Bellman equation defines the optimal value for each state, computing these values on the fly is challenging due to the equation’s recursive nature. Instead, one class of methods used to approximate these values is model-free reinforcement learning, where agents are assumed to update their predictions about the value of each state by comparing their prior expectations about the value of a state with the value actually encountered (i.e., computing a ‘prediction error’). In Temporal Difference (TD) learning models, this prediction error is given by the equation:


2 \[
{\delta _t} = \,{R_{t + 1}} + \gamma \cdot {V_{t + 1}} - {V_t}
\]

i.e., the difference between the original predicted value of the current state (*V*_t_) and the sum of the reward received at the next state (*R*_t__+1_) and the (discounted) value of the new state (*γ* ∙ *V*_t__+1_). Subsequently, the agent’s predicted value of the state is updated using the equation:


3 \[
{V_t} \leftarrow {V_t} + \alpha \left( {{\delta _t}} \right)
\]

where 0 < *α* ≤ 1 is the learning rate. Here, the arrow “←” indicates that a new value is being assigned to the original estimate of *V*_t_.

#### Evidence for Dopamine’s Role as an RPE Signal

Strikingly, research suggests a strong correspondence between TD reward prediction errors and phasic dopaminergic activity in the midbrain (Hollerman & Schultz, 1998; Schultz et al., 1997). These dopaminergic neurons send widespread projections to regions including the striatum, amygdala, and cerebral cortex, which make them particularly well-suited for broadcasting a reward prediction error (RPE) signal across the brain. Indeed, optogenetic studies in Rhesus monkeys have suggested a causal role for dopaminergic activity in reward learning in ways consistent with the RPE hypothesis (Stauffer et al., 2016). RPE-like signals have been most commonly observed in the ventral tegmental area (VTA; which primarily projects to the ventral striatum/nucleus accumbens) and the substantia nigra pars compacta (SNc; which primarily projects to the dorsal striatum) (e.g., Bayer & Glimcher, 2005; Matsumoto & Hikosaka, 2009; Schultz et al., 1997). The presence of widespread dopaminergic projections from the midbrain have led some to suggest that dopamine release serves as a “global reinforcement signal” that strengthens representations associated with reward across the brain (Schultz, 1998). Nevertheless, reward processing and reinforcement learning are more classically associated with the VTA and the ventral striatum, as opposed to the SNc and the dorsal striatum which is more typically associated with a role in movement (Haber, 2014), though recent evidence suggests that this dichotomy may not be as clear-cut as it seems (e.g., Saunders et al., 2018).

In humans, pharmacological manipulations of dopamine levels have also been shown to influence the reward learning process. For example, Pessiglione et al. (2006) conducted an fMRI study in which participants who were treated with either L-DOPA (a metabolic precursor of dopamine) or haloperidol (a D_2_ antagonist) performed an instrumental learning task. Consequently, they found that participants treated with L-DOPA had striatal RPE signals of higher magnitudes and were more likely to choose the most rewarding action than participants treated with haloperidol, suggesting that dopamine plays a causal role in learning through RPEs.

#### Links to Depression

The idea that dopamine serves as an RPE signal offers one possible account for how dopamine dysregulation could lead to depressive symptoms. In particular, a key symptom of depression is anhedonia, or a diminished reactivity to pleasurable stimuli. Anhedonia is often thought to result from reduced primary sensitivity to rewards, which might lead patients to experience less pleasure after reward receipt. This can be formalised computationally using a modification to Equation 2:


3 \[
{\delta _t} = \rho \cdot {R_{t + 1}} + \gamma \cdot {V_{t + 1}} - {V_t}
\]

where *ρ* is the reward sensitivity. Alternatively, anhedonia may also result in a diminished ability to learn from rewards, which may be formalised computationally using the learning rate parameter *α* in Equation 3.

Notably, changes in reward sensitivity and learning rate parameters produce somewhat similar behavioural effects and can sometimes be hard to separate, especially in small behavioural datasets. Nevertheless, they are theoretically distinguishable—in particular, a change in reward sensitivity would affect the asymptotic value of value estimates, while a change in learning rate affects the rate at which value estimates approach this asymptote (Huys et al., 2013). Consequently, Huys and colleagues conducted a meta-analysis in which they fit RL models onto six datasets from probabilistic reward tasks involving 392 experimental sessions and found that MDD and anhedonia affected reward learning more by reward sensitivity parameters than by affecting the learning rates. Nevertheless, care must be taken in drawing conclusions from parameter fits, as parameters often fail to generalise across tasks and models and should hence be interpreted within the context of the specific task and model (Eckstein, Master, et al., 2021; Eckstein, Wilbrecht, et al., 2021).

If depressed individuals had reduced reward sensitivity, one would also expect that they would exhibit attenuated striatal RPE signals in response to rewards. Indeed, Gradin et al. (2011) found that depression was associated with reduced RPE signals in the striatum and midbrain, with anhedonia severity correlating with the degree of signal reduction in several areas, including the nucleus accumbens and midbrain. Moreover, Kumar et al. (2018) further found that MDD individuals showed not just blunted striatal RPE signals but also reduced VTA-striatal connectivity to feedback. Another study by Dombrovski et al. (2015) suggested late-life depression was associated with blunted RPE signals and functional connectivity between the striatum and prefrontal cortex, and these associations were not simply an effect of poor executive control. Additionally. a study conducted by Ubl et al. (2015) suggests that the attenuation in prediction-error signals may be valence-specific, with depressed participants exhibiting an absence of reward-related prediction error signals but increased loss-related prediction error signals in the ventral striatum.

However, not all studies have found a direct link between depression and RPE striatal signals. For example, Greenberg et al. (2015) found that depression was not associated with attenuated striatal RPE signals but instead with an altered relationship between prediction error-related and reward expectancy-related expectancy activity in the ventral striatum. Similarly, Brown et al. (2021) found that depression diagnosis and symptom measures were not associated with differences in striatal reward prediction error signals or expected value signals in the ventromedial prefrontal cortex, though anhedonia moderated the association between RPE and expected value signals. Moreover, Rutledge et al. (2017) found in an fMRI study that depression was associated with neither attenuated striatal RPE signals nor altered associations between RPEs and happiness ratings during a risky decision-making task that did not involve learning. Even amongst studies that have found an effect, results seem to be mixed: for example, in Kumar et al.’s (2018) study, whole-brain analyses failed to reveal any differences between controls and depressed patients, while ROI and connectivity analyses only turning up differences in the right (but not left) striatum. Evidently, the evidence for deficits in RPE signalling in depression is far from unequivocal, and caution must be taken when interpreting positive findings given possible file-drawer effects in decisions to publish.

The tentative association between depression and reduced reward sensitivity/altered RPE signalling, suggests that antidepressants, especially those that directly modulate dopaminergic activity, might alleviate depressive symptoms by reversing these reward-related deficits. However, the evidence for this is mixed at best. For example, Admon et al. (2017) conducted a study in which depressed participants and healthy controls received either a placebo or a low dose of amisulpride, a D_2_/D_3_ receptor antagonist that is thought to increase dopamine signalling through presynaptic autoreceptor blockade. Compared to depressed patients who received a placebo, depressed patients who received amisulpride did indeed exhibit increased striatal activation and corticostriatal functional connectivity in response to rewards. However, amisulpride did not modulate the behavioural impairments in reward learning exhibited by depressed patients. Admon and colleagues hence reasoned that although amisulpride has acute effects on neural function, behavioural modifications might emerge only after sustained administration.

Similarly, Walsh et al. (2018) conducted a study in which depressed patients and healthy controls received bupropion (an NDRI) over six weeks. Consequently, they found that bupropion actually exacerbated reward learning deficits early on in treatment and had positive effects on reward learning only after six weeks of sustained treatment. Walsh and colleagues suggested that the initial exacerbation of impaired learning may have been due to a paradoxical decrease in synaptic dopamine levels following an acute inhibition of dopamine reuptake that was desensitised with repeated treatment, or a decrease in sensitivity in the dopamine reward system due to a decrease in phasic firing of dopamine neurons.

Moreover, Whitton et al. (2020) conducted an fMRI study in which depressed patients completed a probabilistic reward task before and after 6 weeks treatment with pramipexole (a dopamine agonist). Before treatment, the depressed group exhibited lower reward learning than controls. However, although symptoms did improve in the pramipexole treated depressed group, there was no change in reward learning after treatment. Hence, this study once again fails to provide evidence that response to dopaminergic antidepressants is mediated by a remediation in reward learning deficits. Notably, however, Whitton et al.’s study did not include a placebo control group, hence limiting any interpretation of their results.

Interestingly, one recent pharmacological study in anhedonic patients that did observe a change in reward learning accompanied by an alleviation of symptoms involved not a dopaminergic antidepressant, but a kappa-oploid receptor (KOR) antagonist (Krystal et al., 2020; Pizzagalli et al., 2020). Specifically, in a sample of patients with anhedonia and a mood or anxiety disorder, an 8-week treatment with a KOR antagonist resulted in lower anhedonic symptoms, accompanied by improved reward learning on a probabilistic reward task and higher reward-related activation in the ventral striatum compared to a placebo control. Moreover, computational modelling suggested that the treatment led to elevated learning rates, but unaltered reward sensitivity. Consequently, this study serves as a prime example for how reinforcement learning models may help identify the specific computations altered by pharmacological treatments to evaluate the feasibility of novel potential antidepressants.

Overall, neuroimaging studies in patient groups have provided only inconsistent evidence for deficits in RPE signalling in depression, while pharmacological studies in patient groups suggest that dopaminergic antidepressants have, at best, only delayed effects on reward sensitivity. This inconsistent evidence may be due to theoretical and methodological concerns: for example, the diagnostic hetereogeneity inherent in our definitions of depression may preclude us from identifying a single biological phenotype (e.g., Zimmerman et al., 2015), while the experimental paradigms and methods used in these studies may not have been suitable for reliably isolating individual differences (e.g., individual differences in response to treatments; Bolger et al., 2019). Alternatively, another suggestion may be that depression may not be associated directly with deficits in model-free RPE signalling, and that attenuated RPE signals observed in depressed patients in some studies may instead be a result of other deficits. For example, while many studies that have found differences in RPE signalling have involved learning tasks, Rutledge et al. (2017) failed to find any differences in RPE signals on a non-learning task, leading them to suggest that depression may instead involve a deficit in goal-directed decision making and model-based reasoning rather than a primary deficit in model-free RPE signalling.

### Dopamine and Effort-Based Computations

#### Computational Background

While RL tasks typically involve choices between actions that do not differ in effort costs, real-life decisions often necessitate choices about how much effort to dedicate to a particular course of action. In the animal behaviour literature, this effort cost calculation has been modelled by extending RL models to consider how animals choose not just actions, but also the latencies (*τ*) at which to perform these actions (Niv et al., 2007). Given the assumption that animals aim to optimise the long-run average rate of net utility (i.e., rewards - incurred costs) per unit time, vigour selection is critically determined by the average net reward per unit time *R̅*. This is because, for any given latency at which the animal chooses to perform an action, the opportunity cost of this commitment would be *τR̅*, as the animal would be forgoing this much reward on average by completing the chosen action and no other action. In choosing actions and latencies, the animal has to take into account this opportunity cost, along with the cost of performing actions more vigorously and the benefit of obtaining rewards sooner. Consequently, the optimal latency of actions is inversely proportional to the average reward rate.

#### Evidence for Dopamine’s Role in Effort-Based Computations

Given the important role of average reward in determining the optimal vigour of actions, Niv et al. (2007) suggest that tonic dopamine may serve as a slowly changing average reward signal that exerts control over response vigour. This is consistent with experimental results suggesting that higher levels of striatal dopamine lead to enhanced responsivity (Carr & White, 1987; Jackson et al., 1975), while dopamine depletion or antagonism reduces rates of responding (Aberman & Salamone, 1999; Sokolowski & Salamone, 1998). The computational framework set out by Niv et al. also explains why dopamine lesions seem to have minimal effects on low fixed-rate schedules but severely reduce responding on high fixed-rate schedules (Salamone & Correa, 2002).

In support of this idea, pharmacological studies in humans also suggest that manipulating dopamine levels modulates the vigour at which human participants perform actions. For example, Beierholm et al. (2013) conducted a study in which participants received a placebo, L-DOPA, or citalopram (an SSRI) while they performed a rewarded odd-ball discrimination task. Consistent with the framework set out by Niv et al. (2007), Beierholm and colleagues found that higher average reward rates on the task were associated with shorter response times (i.e., higher vigour). Importantly, this relationship between average reward rates and vigour was stronger in the L-DOPA group than in the placebo group, suggesting that dopamine has a causal influence on reward-related vigour. Conversely, citalopram had no effect on the impact of average reward rates on vigour.

While earlier computational work in animals had focused mainly on motor vigour or physical effort, recent work has also linked dopamine to cognitive effort cost calculations. For example, Westbrook et al. (2020) found using positron emission tomography (PET) that participants with higher dopamine synthesis capacity in the caudate nucleus were more willing to perform a harder N-back working memory task for additional money. Moreover, methylphenidate (an NDRI) and sulpiride (a selective D_2_ receptor antagonist that can increase striatal dopamine release at low doses) also increased cognitive motivation for participants with low, but not high, dopamine synthesis capacity. Computational modelling suggested that higher dopamine synthesis capacity and methylphenidate strengthened the influence of benefits of exerting additional effort (i.e., the potential rewards to be gained), suggesting that dopamine modulates decisions about cognitive effort through the perception of its benefits.

Overall, there is convincing evidence to suggest that dopamine might be involved in decisions about exerting both physical and cognitive effort. While mesolimbic pathways between the midbrain and ventral striatum appear important for reward processing, evidence suggests that effort processing may be subserved by a separate mesocortical pathway projecting from dopaminergic midbrain regions to the dorsomedial prefrontal cortex or anterior cingulate cortex (Hauser et al., 2017). Nevertheless, given the complexity and variety of cost-benefit trade-offs involved in decision-making, it is also likely that dopamine signalling in different subregions are important for different computations or types of trade-offs (Westbrook et al., 2021).

#### Links to Depression

Evidence linking dopamine to effort-based computations offers yet another way to account for depressive symptoms like anhedonia. In particular, some researchers have argued that anhedonia may reflect not just reduced responsivity to reward, but also diminished motivation to pursue rewards (Treadway & Zald, 2011). In support of this, Treadway et al. (2012) conducted a study in which MDD patients and healthy controls performed a task where they were required to choose between different difficulty levels associated with varying levels of monetary reward. Consequently, they found that compared to healthy controls, MDD patients were less willing to expend effort to earn larger monetary rewards and showed lower sensitivity to information about reward magnitude and probability of wins when making their choices.

Further evidence for the importance of effort-based decisions in depression comes from a longitudinal study conducted by Berwian et al. (2020). In this study, healthy controls and patients in remission from MDD in response to antidepressants performed an effort task in which they had to choose how much effort to exert (in terms of repeated button presses) to earn varying levels of reward. By modelling their behavioural data using drift-diffusion models (DDM), Berwian and colleagues showed that depressed patients displayed higher mean effort sensitivity than controls, while patients who went on to relapse after the discontinuation of antidepressants required more evidence than non-relapsers when making decisions for low-effort choices.

Overall, these results suggest that effort-related computations play an important role in depression. Consequently, one might expect that antidepressants, especially those that directly modulate dopaminergic function, work by remediating these effort-related deficits, such as by increasing patients’ willingness to exert physical or cognitive effort in a way similar to the effects of dopaminergic manipulations observed in Beierholm et al.’s (2013) and Westbrook et al.’s (2020) studies. However, few studies have explicitly studied these effects in depressed patients treated with antidepressants and related these effects to patients’ responses to antidepressant treatment.

### Dopamine and Model-Based Reinforcement Learning

#### Computational Background

Two distinct learning systems have been described in the RL literature: a computationally cheap model-free system and a computationally expensive model-based system. Model-free RL (as exemplified by TD learning) occurs when reward expectations are formed through direct experience with the environment through reward prediction error computations without the need for a detailed model of the environment, while model-based RL involves forming a model of the environment that incorporates information about transitions between states.

The advantage of model-based over model-free RL algorithms comes from their flexibility: for example, when a local area of the environment changes, model-based RL agents require only a small amount of experience to adapt to these changes, while model-free algorithms will require the agent to relearn the entire value function. However, model-based algorithms are also much more computationally expensive than model-free algorithms. Consequently, people tend to prefer model-free over model-based computations when computational resources are scarce (Otto et al., 2013) or when accuracy is poorly incentivised (Kool et al., 2017).

#### Evidence for Dopamine’s Role in Model-Based Reinforcement Learning

Given dopamine’s role in willingness to expend cognitive effort, one suggestion might be that dopamine also modulates the degree of model-based control used in making decisions. In support of this, Wunderlich et al. (2012) conducted a study in which participants were treated with either Madopar (containing L-DOPA and benserazide) or a placebo before completing a two-step decision task which dissociates model-free and model-based strategies. Wunderlich and colleagues found participants in the L-DOPA group were more likely to take transition probabilities into account when making decisions to stay or switch from first-stage choices, suggesting that they were more reliant on model-based strategies. By fitting computational models on the behavioural data, Wunderlich and colleagues further showed that there was a significant increase in the relative degree of model-based over model-free control in the L-DOPA compared to the placebo group. Similar results were also obtained by Sharp et al. (2016), who found that Parkinson’s disease patients who were off their dopamine replacement medication showed impairments in model-based learning compared to healthy controls that were remediated by dopaminergic medication. In both studies, L-DOPA seems to increase the level of model-based control (i.e., the influence of the interaction between transition structure and reward on decisions) without affecting the level of model-free control (i.e., the influence of reward directly on decisions). From a neuroanatomical standpoint, evidence has implicated the lateral prefrontal cortex (lPFC) in controlling the relative influence of the model-free and model-based systems (Lee et al., 2014; Smittenaar et al., 2013; Weissengruber et al., 2019), while higher presynaptic dopamine in the ventral striatum is associated with greater model-based control and enhanced encoding of model-based information in the lPFC (Deserno et al., 2015).

Besides the arbitration between model-free and model-based systems, researchers have also recently suggested a role for dopamine within model-based learning itself. Although phasic dopamine has traditionally been thought to broadcast an RPE signal that is important mainly for model-free but not model-based RL, recent evidence suggests that these dopaminergic ‘RPE’ signals may also be influenced by model-based computations (Langdon et al., 2018). For example, dopaminergic neurons seem to respond not just to unexpected changes in scalar reward values, but also unexpected changes in other dimensions, such as the flavour of reward pellets, even when there is no change in subjective value (Takahashi et al., 2017). In fact, optogenetic studies in animals suggest that dopamine transients may be necessary for the learning of model-based information, such as the sensory features of rewards (Chang et al., 2017) or transitions between non-rewarding events (Sharpe et al., 2017). Overall, evidence suggests a role for dopamine in model-based learning of non-reward information—however, it is not clear how model-based computations would be affected by the administration of dopaminergic drugs. Historically, researchers have hypothesised that dopamine modulates ‘goal-directed’ (roughly corresponding to model-based) behaviours through projections to the prefrontal cortex and dorsomedial striatum (Balleine & O’Doherty, 2010). However, recent evidence has suggested that the ventral striatum might be important not just for model-free RPE signals but also model-based learning (Daw et al., 2011; Huang et al., 2020), suggesting some overlap between neural systems for model-free and model-based learning.

#### Links to Depression

The distinction between model-based and model-free RL has also been a recent focus in computational psychiatry. In particular, researchers have suggested that an over-reliance on model-free learning might lead to inflexible behaviour and account for the self-regulatory deficits observed across a wide range of disorders (Huys et al., 2015).

Early studies looking into over-reliance on model-free learning in psychopathology had mostly linked these deficits to obsessive-compulsive disorder, eating disorders and addiction, but not mood disorders like depression (Gillan et al., 2016). However, recent studies have suggested that in depressed individuals, these deficits may only be apparent in times of stress. In particular, Heller et al. (2018) conducted a study in which participants completed symptom measures of depression and anxiety followed by a two-step decision-making task. Heller and colleagues found that participants who reported higher levels of depression behaved in a less model-based way after (but not before) a stress induction. Consequently, this deficit in flexible decision-making may impair depressed individuals’ ability to successfully navigate their environment and engage in self-regulation during times of stress. Given the finding that dopamine seems to encourage model-based over model-free behaviour, one might expect that antidepressants, especially those that modulate dopaminergic function, might remediate this over-reliance on model-free learning under stress. However, no study as of yet has directly investigated this possibility.

As mentioned previously, some evidence suggests that depression may not be associated with reduced model-free dopaminergic RPE signalling (e.g., Rutledge et al., 2017). Indeed, evidence for a primary, model-free deficit in depression is equivocal at best. For example, while the studies mentioned above investigating striatal RPE signals in depressed patients had identified differences between patients and controls in the nucleus accumbens, which is thought to be involved in both model-free and model-based computations, there is less consistent evidence for differences in the midbrain dopaminergic nuclei (e.g., Gradin et al., 2011; Kumar et al., 2018), which is typically thought to be more centrally involved in model-free processing (Huys, Daw, et al., 2015). Consequently, Huys, Daw, et al. (2015) suggest that depression may depend not on deficits in model-free computations but on inappropriately low model-based evaluations. Given dopamine’s possible role in model-based learning, one could suppose that any differences in model-based evaluations in depressed patients might be influenced by antidepressants that modulate dopaminergic function. However, the exact ways by which dopaminergic antidepressants could modulate model-based computations are not yet clear.

## What is the Role of Serotonin in Reinforcement Learning?

### Serotonin and Aversive Processing

#### Computational Background

Compared to dopamine, serotonin’s role in RL has not been as well-characterised. Behaviourally, agonising serotonin seems to oppose the behaviours that are activated by dopamine (Fletcher et al., 1993, 1995; Fletcher & Korth, 1999). Moreover, evidence suggests that the serotonin system inhibits dopaminergic function at various levels, including central nulcei such as the VTA and substantia nigra and their terminal sites such as the nucleus accumbens and striatum (Kapur & Remington, 1996).

Consequently, Daw et al. (2002) suggest that serotonin may act as a motivational opponent to dopamine in reinforcement learning. Specifically, phasic serotonin may report a ‘punishment prediction error’ signal, much like dopamine is thought to broadcast a ‘reward prediction error’ signal. Moreover, given that tonic dopamine might report an ‘average reward signal’ that determines effort or response vigour (Niv et al., 2007), Cools et al. (2011) suggest that tonic serotonin may similarly report the average rate of punishment. Following Niv et al.’s logic about higher average reward rates invigorating action, higher average rates of punishment (possibly reported by higher tonic serotonin levels) could instead lead to lower vigour when actions are more likely to have aversive outcomes. Based on the observation that the dorsal (and not the median) raphe nuclei projects to areas also innervated by the dopaminergic system like the striatum, supporters of the opponency account have suggested that it is the dorsal raphe serotonin projections that are involved in aversive processing and ‘opposing’ dopamine (Daw et al., 2002).

#### Evidence for Serotonin’s Role in Aversive Processing

These ideas about serotonin are broadly consistent with earlier ideas about serotonin as playing a role in aversive processing and behavioural inhibition. In particular, based on evidence that serotonergic manipulations affect the avoidance responses of animals in response to aversive stimuli, Deakin and Graeff (1991) proposed that serotonin plays an important role in adaptive responses to aversive stimuli. Specifically, different serotonergic projections from the dorsal raphe nucleus (DRN) may facilitate different responses depending on the spatiotemporal distance of the aversive outcome, with projections from the DRN to the amygdala facilitating anticipatory anxiety to distal threats, while projections to the PAG facilitating a flight/fight response or panic responses to more proximal threats (Deakin & Graeff, 1991; Paul & Lowry, 2013). On the other hand, based on the observation that blockade of serotonergic transmission disinhibits behaviours that are punished by aversive outcomes (Harrison et al., 1997, 1999), Soubrié (1986) instead proposed that serotonin plays a role in behavioural inhibition. Importantly, these ideas about serotonin may not be mutually exclusive and are broadly consistent with the proposed roles of phasic serotonin as reporting a punishment prediction error and tonic serotonin as reporting an average punishment signal that inhibits behavioural vigour.

However, despite broad evidence for these computational ideas, recordings of serotonergic neurons have not yet uncovered signals with a precise computational interpretation like those of dopaminergic neurons. This might be because before the advent of optogenetic techniques, targeting of serotonergic neurons had not reached a degree of precision similar to the targeting of dopaminergic neurons (Cools et al., 2011). Nevertheless, pharmacological manipulations in humans reveal a role for serotonin that is broadly consistent with these computational ideas. For example, Cools et al. (2008) conducted a study in which participants who underwent acute tryptophan depletion (ATD) or control treatment performed a reversal learning task where they were asked to predict whether a stimulus would lead to a reward or a punishment. Consequently, they found that participants made more errors for punishment-associated stimuli than for reward-associated stimuli under baseline, but ATD enhanced the ability to predict punishments without affecting the ability to predict rewards. Although the promotion of aversive processing from ATD may seem at odds with the idea oof serotonin as an aversive prediction error, Cools and colleagues suggest that the depletion of tonic serotonin might have enhanced the dynamic range of phasic serotonin activity, hence increasing the signal-to-noise ratio of phasic serotonergic signals. Such an effect would be consistent with findings and models in other neurotransmitters such as dopamine (Grace, 1991, 2000) and noradrenaline (Aston-Jones et al., 1999) where tonic neurotransmitter levels modulate the level of phasic responses. Hence, Cools et al.’s results may provide some evidence the role of phasic serotonin in reporting aversive prediction errors. However, this evidence is rather indirect, and direct evidence for a role for serotonin in reporting aversive prediction errors is still lacking.

Moreover, support for a role of serotonin in inhibiting motor vigour comes from another pharmacological study conducted by Crockett et al. (2009). In this study, participants performed a version of a go/no-go task where go and no-go responses were differentially rewarded or punished. Following placebo treatment, participants were slower to respond in punishment conditions compared to in reward conditions. However, ATD treatment abolished this effect of punishment-related inhibition despite no change in overall motor response inhibition, consistent with the idea of serotonin as playing a role in punishment-related behavioural inhibition.

Besides punishments, serotonin may also be involved in the processing of other costs of actions, such as effort. For example, while dopamine may be involved in assessing the benefits of exerting additional effort (e.g., the prospect of additional reward), serotonin may be involved in assessing the costs of effort exertion. Indeed, Meyniel et al. (2016) found that healthy participants who underwent an 8-week escitalopram (an SSRI) treatment produced more effort on a handgrip force production task than participants who received a placebo treatment. Furthermore, their computational model revealed that SSRI treatment seemed to affect decisions about effort by reducing participants’ assessment of effort costs rather modulating their sensitivity to monetary incentives, suggesting a role for serotonin in effort-based computations that may be complementary to that of dopamine.

#### Links to Depression

If learning about rewards and punishments are subserved by different systems, one idea might be that depression is marked not just by a reduced sensitivity to reward (possibly reflected by attenuated dopaminergic RPE signals as reviewed above), but also a heightened sensitivity to punishment. Indeed, Dombrovski et al. (2013) found that on a probabilistic reversal learning task, MDD patients were more likely to switch after misleading negative feedback (i.e., receiving a punishment even though the contingency of the stimuli had not changed) than controls, suggesting that MDD patients were oversensitive to punishments.

However, if serotonin is associated with greater aversive processing and behavioural inhibition, the question remains over why depression, which is characterised by increased aversive processing and reduced behavioural vigour, is treated by increasing (and not decreasing) serotonergic levels through the administration of antidepressants like SSRIs. One possible answer comes from Michely, Eldar, Erdman et al. (2020), who proposed one way in which enhanced punishment learning (and reduced reward learning) could lead to more positive affective experiences. Michely and colleagues conducted a study in which healthy participants received citalopram (an SSRI) or placebo treatment and performed a gambling card game involving learning about the likelihoods of gambles succeeding. By fitting RL models on participants’ choices, they found that after a week of treatment, SSRIs reduced the learning rates for reward but enhanced learning rates for punishments. Michely and colleagues suggest that this asymmetric effect of SSRIs on learning rates eventually leads to lowered positive expectations and enhanced negative expectations, which in turn leads to stronger positive surprise and attenuated negative surprise when rewards or punishments are received. Since positive and negative surprise has been shown to be strongly linked to mood (Rutledge et al., 2014), this increased sampling of positive surprise over time could lead to improvements in mood. In other words, by lowering their expectations, SSRI treatment could eventually cause individuals to perceive events as more rewarding and less disappointing, hence leading to improvements in mood.

Although intriguing, this account has not yet been verified in clinical studies involving depressed patients. Hence, in future studies, it would be interesting to test whether an improvement in depressive symptoms following antidepressant treatment is indeed mediated by the learning asymmetry and lowered expectations induced by these treatments.

### Serotonin and Reward Processing

#### Evidence for Serotonin’s Role in Reward Processing

Despite the elegance of the opponency account, evidence strongly suggests that the roles of dopamine and serotonin may not be as simple as the opponency account suggests. For example, although there is no direct evidence for a role of serotonin in reporting punishment prediction errors, there is actually evidence suggesting that dopaminergic neurons in the ventral tegmental area may report aversive prediction errors (de Jong et al., 2019; Salinas-Hernández et al., 2018), suggesting that the delineation between dopamine and appetitive processing versus serotonin and aversive processing may not be as clear cut as the opponency account suggests. Indeed, evidence also suggests that serotonin may play a role in not just punishment processing but also reward processing. For example, Bromberg-Martin et al. (2010) recorded dorsal raphe neuron activity in macaques while they performed reward-oriented saccade tasks and found that the activity of these neurons seemed to encode information about reward-related cues and outcomes. Similarly, using fibre photometry and single-unit recordings, Li et al. (2016) found that rewards such as sucrose, food, sex, and social interaction rapidly activated presumptive serotonergic neurons in the dorsal raphe nucleus, but aversive stimuli like quinine and footshocks did not. Collectively, these results suggest that serotonin may play a complementary, rather than opposing, role to dopamine in reward and punishment processing.

Indeed, pharmacological studies in humans also implicate serotonin in reward processing. For example, Seymour et al. (2012) conducted a study in which participants who underwent either ATD or placebo treatment performed a probabilistic instrumental learning task involving both appetitive (financial rewards) and aversive (electric shocks) outcomes. Seymour et al. found that participants who experienced ATD had lower reward sensitivity (as suggested by a reduced effect of reward magnitude on decisions to repeat a choice), but were not significantly different in punishment sensitivity compared to the placebo group. Moreover, the ATD group also had attenuated reward-related brain responses in the ventromedial prefrontal cortex and reward prediction error responses in the right dorsolateral putamen, suggesting that serotonin might play a role in the neural representation of reward outcomes. Similarly, Kanen et al. (2021) found that participants who underwent ATD exhibited impairments in instrumental learning compared to placebo controls in conditions that involved learning about reward or both reward and punishment, but not in the punishment-only condition, suggesting a role for serotonin in learning appetitive responses. In the social domain, Frey and McCabe (2020) conducted an experiment in which participants learnt the association between name cues and rewarding or aversive social outcomes (happy or fearful faces respectively) and found that participants who underwent ATD exhibited impaired social reward learning and altered social reward prediction signals in the insula, temporal lobe, and prefrontal cortex compared to placebo controls.

Overall, there is substantial evidence for serotonin’s involvement in reward processing. Indeed, serotonergic neurons in the dorsal and median raphe nucleus project to a wide variety of brain areas associated with reward processing, including the mesolimbic dopamine system (the ventral tegmental area and nucleus accumbens), the lateral habenula, parabrachial nucleus, medial prefrontal cortex, and orbitofrontal cortex (Kranz et al., 2010). Proponents of the opponency account had interpreted these projections as evidence for serotonin’s role in ‘opposing’ the reward-related effects of dopamine—however, given the increasing body of evidence for a role for serotonin in reward processing, it is possible that these projections are also directly involved in the processing of reward and not just aversive outcomes.

#### Links to Depression

As reviewed above in the sub-section on dopamine and reward processing, symptoms of depression like anhedonia could be thought of as stemming from a reduced sensitivity to reward. Consequently, if serotonin does indeed play a role in reward processing, one might postulate that serotonergic antidepressants might also alleviate depressive symptoms by remediating these reward-related deficits.

While few studies have directly studied this possibility in depressed patients, some studies have found evidence that SSRIs modulate reward processing in healthy participants. For example, Scholl et al. (2017) conducted an fMRI study in which participants received either 2 weeks of citalopram or placebo treatment while they performed a task in which they had to concurrently learn about pleasant (amount of monetary reward) and unpleasant (amount of effort) outcomes. They found that citalopram administration enhanced neural learning signals, including both reward prediction error signals in the ventromedial prefrontal cortex (vmPFC) and effort prediction error signals in the dorsal anterior cingulate (dACC), hence suggesting a role for serotonin in the learning of both pleasant and unpleasant outcomes. Behaviourally, citalopram treatment also led to reward learning that was more robust to negative interference from irrelevant factors (e.g., the absence of real reward experience). Overall, these results suggest a role for serotonin in not just learning about aversive outcomes (as suggested by the opponency account), but also about rewards.

Alternatively, evidence suggests that there may be bi-directional interactions between mood and reward processing, where mood both depends on reward outcomes and biases how people perceive reward outcomes (Eldar et al., 2018; Eldar & Niv, 2015). Consequently, Eldar et al. (2016) propose that mood may serve as a representation of the ‘momentum’ of reward outcomes that biases our perception of future reward outcomes in a way that helps us account for environmental dependencies. Accordingly, Michely, Eldar, Martin, et al. (2020) suggest that rather than influencing reward processing directly, SSRIs may have an effect on interactions between mood and reward processing. In their study, participants who received a week-long citalopram or placebo treatment performed two blocks of a reward learning task on each day. Between each block of the task, participants played a Wheel of Fortune (WoF) game designed to have a strong effect on participants’ affective states where they either won or lost a significant amount of money. Consequently, they found that the WoF mood induction affected reward perception, with participants later showing a preference for images they encountered when they were in a better mood, i.e., after a WoF win or before a WoF loss. This mood learning bias was modulated by citalopram administration, with the citalopram group showing an enhanced positive, but not negative, mood bias compared to the placebo group. Fitting a computational model with positive and negative mood bias parameters similarly suggested that citalopram increased positive (but not negative) mood bias compared to controls. Consequently, Michely and colleagues suggest that SSRIs lead to gradual improvements in mood by creating a positive feedback loop between reward and mood where the boosted perception of rewards leads to further improvements in mood, which then further boosts the perception of reward.

Overall, evidence seems to suggest that SSRIs affect not just aversive processing, but also reward processing (either directly or indirectly via the modulation of mood-reward interactions). However, these findings have not yet been replicated in samples of depressed patients undergoing antidepressant treatment. Moreover, the idea of SSRIs as strengthening a positive mood bias on reward perception seems somewhat at odds with Michely, Eldar, Erdman et al.’s (2020) proposal of SSRIs as enhancing punishment learning and dampening reward learning. Consequently, more work would be necessary to reconcile these contrasting accounts.

### Serotonin and Delay Discounting

#### Computational Background

When making decisions, one needs to take into account not only the immediate rewards that could be obtained but also any future rewards that may become available as a result of that decision. In TD models, future rewards are taken into account via the term *γ* ∙ *V*_t__+1_ in Equation 2, which allows rewards from future states to bleed back in time to their antecedent states. Here, the discounting factor *γ* controls how far into the future the agent takes into account in evaluating values—when *γ* is small, the agent takes into account only short-term rewards, while a large *γ* encourages the agent to also take into account long-term rewards.

#### Evidence for Serotonin’s Role in Delay Discounting

TD models offer a way to computationally formalise Soubrié’s (1986) idea of serotonin as playing a role in behavioural inhibition. In particular, besides inhibiting behaviour to avoid punishments, animals might also need to inhibit their behaviours to ‘wait’ for delayed rewards. Consequently, some have argued that serotonin might modulate the discount factor *γ*, affecting how we evaluate decisions and compute prediction errors (as in Equations 2–3; Schweighofer et al., 2007). Support for this idea comes from animal studies: for example, Wogar et al. (1993) found that rats that received sham lesions were willing to wait longer for larger reinforcers than rats with lesions in the ascending serotonergic system. Similarly, Miyazaki et al. (2011) found that putative serotonergic neurons in the DRN increased their tonic firing rates while rats waited for rewards and stopped firing when they gave up waiting because of extended delays or reward omission, hence supporting a role for serotonin in waiting for delayed rewards.

Pharmacological studies in humans have similarly implicated serotonin in delay discounting. For example, Schweighofer et al. (2008) conducted a study in which participants who underwent tryptophan depletion, tryptophan loading, or a control treatment performed a delayed reward choice task where they had to choose between pursuing an immediate but smaller reward or a delayed but larger reward. Consequently, Schweighofer and colleagues found that participants who underwent tryptophan depletion were more likely to choose immediate small rewards than those who underwent tryptophan loading. Indeed, by fitting an RL model on participants’ choices, Schweighofer and colleagues found that participants in the depletion condition had a lower value of *γ* (i.e., increased delay discounting) than participants in the control or loading conditions. This is consistent with animal studies in supporting the idea that serotonin modulates the discount factor that determines how future rewards are evaluated. Schweighofer et al. (2007) proposes that the serotonin’s modulation of the discount rate could be mediated by serotonergic projections to the basal ganglia (which allows it to modulate the computation of the value function, i.e., the role of *γ* in Equation 1) and serotonin’s ability to control dopamine release in the striatum (which allows it to modulate the computation of the RPE, i.e., the role of *γ* in Equation 2).

#### Links to Depression

Consequently, one interesting question might be whether depression may be associated with alterations in delay discounting rates that are remediated by serotonergic antidepressants. However, few studies in depressed patients have studied delay discounting using the experiential delay discounting paradigm (i.e., forcing the agent to wait for delayed outcomes) that is used in animal studies of delay discounting and in Schweighofer et al.’s (2008) study in humans. Instead, many studies in depressed patients involve economic decisions in which time intervals are presented verbally or symbolically to participants. It is important not to treat these two paradigms as interchangeable as they do not necessarily tap on the same mechanisms: for one, experiential delay discounting paradigms (but not verbal economic decision-making paradigms) actually involve learning and can be described using RL models. However, since there is some initial evidence that delay preferences in humans are reliable across both verbal and experiential delay tasks (Lukinova et al., 2019), we will still discuss the results of these studies to determine what, if anything, they tell us about delay discounting in depression.

Given that depression is characterised by hopelessness about the future, Pulcu et al. (2014) suggested that depressed individuals might use more short-term economic decision-making strategies and exhibit increased delay discounting. In support of this, Pulcu and colleagues conducted a study in which depressed patients, remitted depressed patients, and healthy controls performed a delay discounting task involving monetary rewards. Consequently, they found that depressed patients had higher discounting rates for large rewards relative to healthy and remitted participants. In line with these results, Amlung et al. (2019) conducted a meta-analysis of delay discounting studies and found steeper discounting in patients with MDD.

Given these results, a natural conclusion might be that depression is associated with increased discounting rates and that serotonergic antidepressants like SSRIs could alleviate depressive symptoms by decreasing the discounting rate, leading depressed individuals to pursue more long-term economic decision-making strategies. However, few studies have directly studied this possibility in depressed patients undergoing SSRI treatment. Moreover, there is also conflicting evidence on the association between depression and delay discounting. For example, Lempert and Pizzagalli (2010) found that anhedonia was actually associated with decreased discounting rates. Moreover, by fitting multilevel models on participants’ choices on a delay discounting task, Tsypes et al. (2022) found that suicidal ideation and behaviour were associated with inconsistent reward valuation (i.e., a parameter that controlled participants’ sensitivities to the relative expected values of the available options when making their choices) rather than a difference in delay discounting (i.e., the discount rate parameter). Overall, further studies are required to establish whether depression and antidepressants truly alter patients’ discounting rates.

## Discussion

Overall, we have surveyed a broad range of ideas about the roles of neurotransmitters in reinforcement learning and discussed the possible implications of these ideas for our understanding of antidepressants. These roles include roles for dopamine in reward processing, effort-based computations, and model-based reinforcement learning; and roles for serotonin in aversive processing, reward processing, and temporal discounting. The evidence implicating these roles in depression and antidepressant treatment is summarised in Table 1 below.

**Table 1:** Table summarising the evidence implicating the various proposed computations performed by dopamine and serotonin in depression and antidepressant treatment.


PROPOSED ROLES OF NEUROTRANSMITTERS IN RL		IS THERE EVIDENCE IMPLICATING THESE PROCESSES IN ANTIDEPRESSANT TREATMENT?			

		EVIDENCE FOR MODIFICATION BY PHARMACOLOGICAL INTERVENTION	EVIDENCE FOR ASSOCIATION WITH DEPRESSION	EVIDENCE FOR MODIFICATION BY ANTIDEPRESSANT TREATMENT IN DEPRESSED PATIENTS	EVIDENCE THAT RESPONSE TO ANTIDEPRESSANT TREATMENT IS MEDIATED BY PROCESS

**Dopamine**	Dopamine as RPE Signal	Yes, e.g., Pessiglione et al., (2006)	Yes for behaviour, mixed neural evidence, e.g., Gradin et al., (2011), Rutledge et al., (2017)	Mixed evidence (Admon et al., 2017; Walsh et al., 2018; Whitton et al., 2020)	–

	Dopamine and Effort-Based Computations	Yes, e.g., Beierholm et al. (2013)	Yes, e.g., Treadway et al. (2012)	–	–

	Dopamine and Model-Based RL	Yes, e.g., Wunderlich et al. (2012)	Yes, but only under stress induction (Heller et al., 2018)	–	–

**Serotonin**	Serotonin and Aversive Processing	Yes, in punishment learning (Cools et al., 2008) and inhibiting vigour (Crockett et al., 2009)	Yes, e.g., Dombrovski et al. (2013)	–	–

	Serotonin and Reward Processing	Yes, in reward learning (e.g., Scholl et al., 2017) and ‘momentum’ (e.g., Michely, Eldar, Martin et al., 2020)	Yes (see dopamine and RPEs)	–	–

	Serotonin and Temporal Discounting	Yes, e.g., Schweighofer et al. (2008)	Mixed evidence, e.g., Pulcu et al. (2014), Lempert & Pizzagali (2010)	–	–


In general, these roles of dopamine and serotonin have been backed by convincing evidence from both animal studies and pharmacological studies in humans. Concurrently, there is also some evidence for depressed patients experiencing deficits in these same aspects of RL compared to healthy controls. Collectively, these lines of evidence hint at the fact that the action of antidepressants may also depend on these same computational mechanisms. However, as evident from the table above, few studies have explicitly studied these processes in depressed patients undergoing antidepressant treatment, and even fewer have directly probed whether response to antidepressant treatment is mediated by these processes. Future studies probing these processes in depressed patients would hence be necessary before making firm conclusions about the processes underlying antidepressant treatment.

In our introduction, we introduced RL models as a potential way to help us study interactions between the individual and the environment. While few studies have explicitly drawn the link between RL models and the environment, some of the existing RL models may offer predictions for how the environment may affect antidepressant action. For example, in Michely, Eldar, Martin, et al.’s (2020) ‘mood-as-momentum’ model, increases in positive momentum as a result of SSRI administration may not lead to marked improvements in mood in environments that are bereft of rewards. Consequently, RL models may account for why antidepressant efficacy differs based on environmental circumstances such as socio-economic status (Viglione et al., 2019)—a possibility that should be explored in future studies.

Moreover, while we have focused mainly on dopamine and serotonin, numerous other neurotransmitters are also implicated in antidepressant mechanisms, including noradrenaline (Dell’Osso et al., 2010), glutamate (Krystal et al., 2013), and GABA (Krystal et al., 2002). However, the roles of these other neurotransmitters in RL is less clear. Future studies looking into the possible roles of these neurotransmitters in RL could help us better understand the mechanisms behind the action of different classes of antidepressants, including novel ‘rapid-onset antidepressants’ like ketamine.

Evidently, although there has been no shortage of studies investigating the relationship between RL processes, we are still a long way from drawing firm conclusions about the involvement of RL processes in response to antidepressant treatment. The table below summarises our recommendations for future research directions:

**Questions for Future Research**

- What are the specific reward- or punishment-related neural computations that are altered in depressed patients? For example, depression may be associated with aberrant responses in the ventral striatum, but the ventral striatum appears to be involved in both model-free and model-based learning. Is depression associated with deficits in model-free learning, model-based learning, or both?
- Are treatment responses to antidepressants mediated by changes in reward- or punishment-related computations? Are improvements in different symptoms associated with changes in different computations (e.g., anhedonia with reward sensitivity or negative affect with aversive processing)? Can these differences be related to different monoaminergic pathways?
- While dopamine and serotonin were initially thought to have different roles, there is increasing evidence that both neurotransmitters may also be involved in similar neural computations (e.g., reward processing through interactions with the striatum). Do antidepressants that modify the function of different neurotransmitters modify the same or different RL computations?
- Do modifications to reward- or punishment-related computations following pharmacological treatment depend on properties of the individuals’ environments (e.g., the abundance or sparseness of rewards)? Can these interactions help account for differential responses of individuals to antidepressant treatment?

## Limitations

In this paper, we have attempted to give a high-level overview of the literature surrounding reinforcement learning models of neurotransmitter function, depression, and antidepressant mechanisms. To do so, we drew upon a wide variety of studies, including pre-clinical animal work, neuroimaging associational studies, pharmacological studies in non-clinical and clinical human participants, etc. The strength of the evidence provided by each study necessarily depends on methodological factors: for example, pharmacological studies in clinical participants tend to have small sample sizes, while computational modelling studies are particularly prone to errors in model fitting and performing sanity checks that compromise their conclusions (Palminteri et al., 2017). The diversity of studies cited prevented us from systematically assessing the included studies against pre-defined quality criteria, while many historically important computational modelling studies do not report metrics (e.g., parameter recovery performance) that are now considered good practice. Nevertheless, the lack of systematic assessment of study quality is an important limitation of our review, and while we have attributed more weight to results only when similar effects have been demonstrated across multiple studies, we also urge readers to exercise caution in drawing conclusions from the results of any individual study without paying close attention to the study’s methodology.

## Conclusion

In conclusion, RL models have contributed greatly to our understanding of neurotransmitter function, leading to a host of ideas about how RL processes may be modified by antidepressant treatment. However, there is still insufficient evidence directly implicating these RL processes in the response of depressed patients to antidepressant treatment. Future studies directly investigating RL processes in depressed patients are hence warranted, especially given the huge potential of RL models to help us better understand the processes underlying antidepressant action and generate insights that inform clinical treatment.
